# The new horizon of liquid biopsy in sarcoma: the potential utility of circulating tumor nucleic acids

**DOI:** 10.7150/jca.42816

**Published:** 2020-07-09

**Authors:** Junqiang Wei, Xinyue Liu, Ting Li, Peipei Xing, Chao Zhang, Jilong Yang

**Affiliations:** 1 Department of bone and soft tissue tumor, Tianjin Medical University Cancer Institute and Hospital, Tianjin, 300060, China; 2 National Clinical Research Center for Cancer, Key Laboratory of Cancer Prevention and Therapy, Tianjin's Clinical Research Center for Cancer, Tianjin's Medical University Cancer Institute and Hospital, Tianjin, 300060, China.; 3 Department of Orthopedics, Affiliated Hospital of Chengde Medical College, Chengde, Hebei, 067000, China

**Keywords:** sarcoma, liquid biopsy, circulating tumor nucleic acids, bloodborne biomarkers

## Abstract

The diagnosis, treatment and prognosis of sarcoma are mainly dependent on tissue biopsy, which is limited in its ability to provide a panoramic view into the dynamics of tumor progression. In addition, effective biomarkers to monitor the progression and therapeutic response of sarcoma are lacking. Liquid biopsy, a recent technological breakthrough, has gained great attention in the last few decades. Nucleic acids (such as DNA, mRNAs, microRNAs, and long non-coding RNAs) that are released from tumors circulate in the blood of cancer patients and can be evaluated through liquid biopsy. Circulating tumor nucleic acids reflect the intertumoral and intratumoral heterogeneity, and thus liquid biopsy provides a noninvasive strategy to examine these molecules compared with traditional tissue biopsy. Over the past decade, a great deal of information on the potential utilization of circulating tumor nucleic acids in sarcoma screening, prognosis and therapy efficacy monitoring has emerged. Several specific gene mutations in sarcoma can be detected in peripheral blood samples from patients and can be found in circulating tumor DNA to monitor sarcoma. In addition, circulating tumor non-coding RNA may also be a promising biomarker in sarcoma. In this review, we discuss the clinical application of circulating tumor nucleic acids as blood-borne biomarkers in sarcoma.

## Introduction

Sarcomas are a heterogeneous group of malignant solid tumors that originate from mesenchymal tissues and encompass more than 70 histological subtypes 1, 2. Sarcomas account for nearly 1% of all adult malignancies and approximately 15%-21% of paediatric malignant tumors 2, 3. Although the 5-year survival rate of malignant bone tumors, such as osteosarcoma (OS), Ewing sarcoma (ES) and chondrosarcoma (CS), has increased to 50%-70% with the advent of effective chemotherapeutic agents, the survival rate has reached a plateau over the past decades 1. The same trend is observed in soft tissue sarcomas 1, 4. The mortality of bone and soft tissue sarcomas in the USA in 2019 is estimated to be as high as 41% and 47%, respectively 3. Based on the data of the “Surveillance of rare cancer in Europe” (RARECARE) project, the five-year relative survival was 58% for soft tissue sarcomas (STSs) and 62% for bony sarcomas in European regions between 2000 and 2002 5. However, there is a paucity of overall sarcoma data from Asia. The National Central Cancer Registry of China estimated that there were 28,000 new bone sarcoma diagnoses and 20,700 deaths (73.9%) from bone sarcoma in China in 2015 6. Approximately 39,900 new STS cases occurred nationwide in China in 2014, but the data on mortality were lacking 7. By analysing data from the Bone and Soft Tissue Tumor (BSTT) registry of Japan from 2006 to 2013, the cumulative disease-specific survival values of bone sarcoma at 2 and 5 years were 80.3% and 70.3%, respectively 8. Existing evidence has shown that racial and ethnic disparities do exist with respect to occurrence and mortality in sarcoma. Studies have revealed that sarcomas are more prevalent in Caucasian descent (58.1%) than Hispanic (21.4%) or African Americans (13.0%), and Hispanic or African descent Americans had significantly poorer overall survival at the 5-years follow-up point than Caucasian descent 9-11. The poor prognosis of sarcoma is partly attributed to a lack of effective biomarkers for early diagnosis, screening, prognosis and tumor monitoring.

At present, the diagnosis, treatment and monitoring of tumors mainly depend on tissue biopsy and imaging examination. Tissue biopsy currently remains the gold standard for diagnosis and therapy 12, 13. However, tissue biopsy is associated with several limitations (Table 1). For example, tissue biopsy is invasive and may result in some complications 14, 15. Furthermore, tissue biopsy only provides a snapshot of the genetic alterations in a single part of the tumor and does not reflect the dynamic changes of the tumor 16, 17. Tumor progression and response to chemotherapy or surgery also rely on radiological imaging investigation, but the imaging methods have limited sensitivity and specificity 17. Distinguishing tissues with residual tumor from tissues affected by inflammatory changes after surgery can be difficult in some cases 18. In addition, the potential side-effects from radiological examination is a health hazard that need to be considered. Therefore, identifying a more effective and non-invasive method that can be used to monitor tumor dynamics is urgently needed.

**Figure 1:**
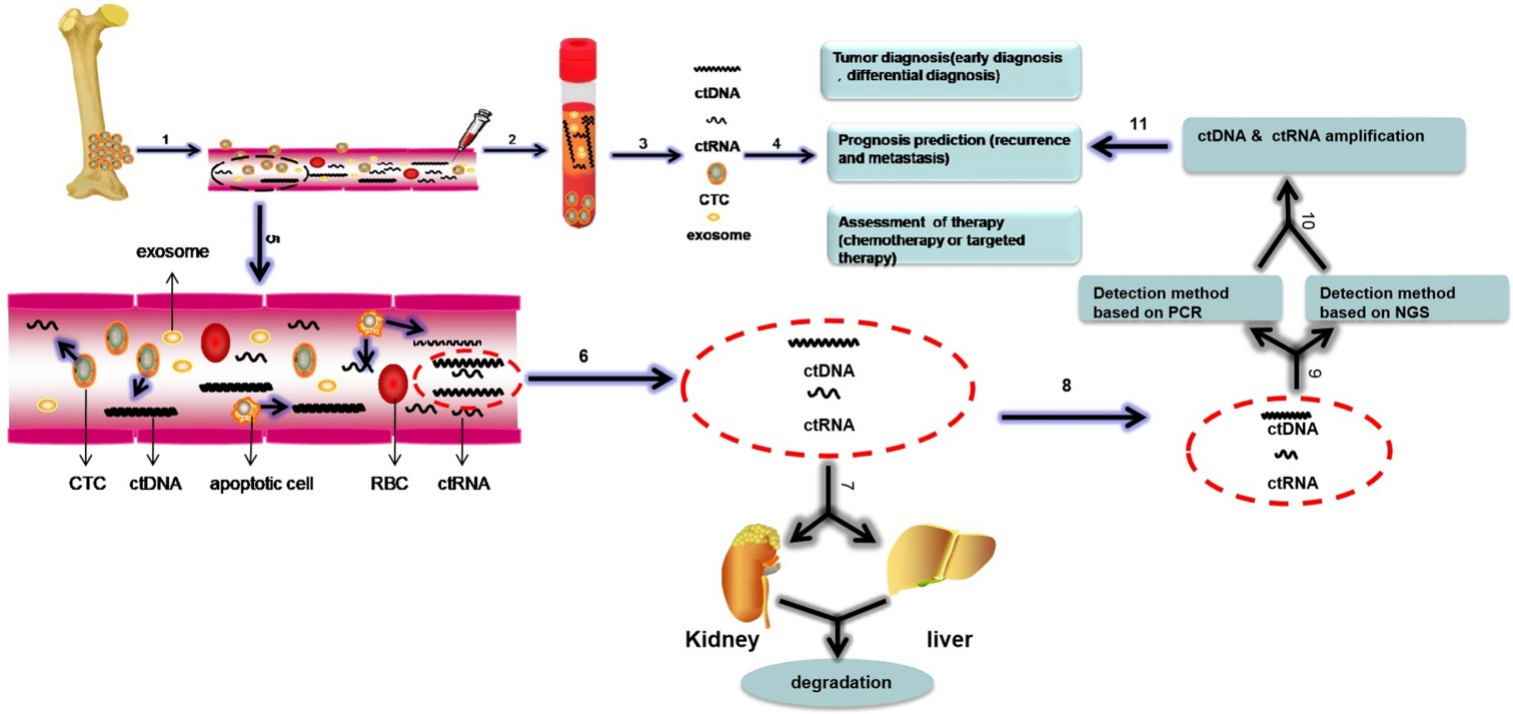
** The biological processes and detection methods of circulating nucleic acids and a schematic of liquid biopsy in sarcoma.** Liquid biopsy includes the isolation of circulating tumor cells (CTCs), circulating tumor DNA (ctDNA) and circulating tumor RNA (ctRNA) and exosomes from the blood. Clinical application of liquid biopsy in sarcoma involve diagnosis in the early stage, monitoring of sarcoma progression, (e.g., local recurrence and distant metastasis), prediction of prognosis and detection of response to therapy. The biological processes of circulating nucleic acids are shown in the magnified view in step 5, and ctDNA, ctRNAs, and exosomes can be discharged into the circulation by apoptotic or necrotic CTCs. Some of the circulating nucleic acids are degraded by the liver and kidney, and the remaining circulating nucleic acids can be detected and amplified by PCR and NGS, which can be used to monitor sarcomas. Bloodborne CTCs, ctDNA, ctRNAs and exosomes can be analysed to diagnose sarcoma, predict prognosis and evaluate response to therapy. This process is called “liquid biopsy”. Based on these steps, we can monitor the tumor. Abbreviations: CTCs: circulating tumor cells; ctDNA: circulating tumor DNA; ctRNA: circulating RNA; RBC: red blood cell

Liquid biopsy has gained great attention in the last few decades as an alternative to monitor malignant neoplasms and was listed as one of the top ten technological breakthroughs in 2015 19. Compared with tissue biopsy, liquid biopsy has several advantages, including a non-invasive procedure, real-time nature and the potential to provide a panoramic molecular portrait of the tumor (Table 1). Although liquid biopsy mainly refers to the monitoring of circulating tumor nucleic acids (ctNAs), it also includes the detection of circulating tumor cells (CTCs) and tumor-derived exosomes 20-22 (Figure 1). A comparison of the three approaches to liquid biopsy is listed in Table 2.

Because of the rarity of sarcoma and its high heterogeneity, the literature on CTCs and tumor-derived exosomes in sarcoma is limited 23, 24. The production of ctNAs is a more frequent event in the course of cancer progression. Studies have estimated that a tumor that weighs 100g is equivalent to 3×10^10^ tumor cells, and 3.3% of tumor DNA will be released into blood circulation per day 25. ctNAs include circulating tumor DNA (ctDNA) as well as circulating tumor RNAs (ctRNAs), such as mRNAs, microRNAs (miRNAs) and long non-coding RNAs (lncRNAs). The dynamic change of these molecules can reflect the pathological process of tumor progression and indicate the presence of a benign or malignant lesion 26. ctNAs can also reflect the profile of the tumor genotype in real-time and with a non-invasive approach compared with tissue biopsy which reflects a single point in time of a single site of the tumor 19.

In this review, we will discuss recent advances in the identification of ctNAs in sarcoma and their potential clinical applications. We will also review the current limitations of ctNAs and the prospects for translation into clinical practice.

## Biology of ctNAs

Current studies have suggested that cell-free DNA (cfDNA) that circulates in the blood is derived from primary tumors, CTCs, distant micrometastatic lesions, normal haemocytes, and normal stromal cells 27. Existing evidence has shown that a primary tumor releases 10^6^ CTCs per gram of tumor every day, the majority of which die, as only approximately 0.01% of CTCs survive 28, 29. ctDNA is the DNA released from tumor cells in cancer patients 21. It is released into the bloodstream during the necrotic and apoptotic processes of tumor cells and metabolized in the liver and kidney (Figure 1). It comprises 0.1% to 90% of cfDNA 30, 31. The half-life of ctDNA varies in different types of tumors and different individuals, ranging from 15 minutes to several hours 21, 30. In addition to ctDNA, other types of ctNAs, including circulating tumor transcripts and non-coding RNAs such as miRNAs and lncRNAs, are also discharged from dying tumor cells into the circulation 26, 32 (Figure 1). Increasing evidence has indicated that circulating tumor transcripts and non-coding RNAs are highly stable 33, 34. They are protected from degradation by their packaging into exosomes, microvesicles or other vesicle types 33-36 or in an EV-independent fashion, and they are detected in complexes with the protein Argonaute 2 (AGO2) 37, 38 or high-density lipoproteins (HDLs) 39 in the extracellular space and body fluids. Therefore, circulating transcripts and non-coding RNAs are stable and can survive in the bloodstream for a long time. However, the half-life of circulating tumor RNAs is not established 40. Although ctNAs reflect the tumor dynamics and tumor burden, the enrichment and detection of such low concentrations of ctNAs in circulation remain challenging.

## Detection Methods for ctNAs

Currently, the two most common methods used to detect ctDNA include polymerase chain reaction (PCR)-based methods and next generation sequencing (NGS)-based methods (Figure 1). Digital PCR (dPCR) is the latest technology and can quantify nucleic acid molecules in tumor samples. This technique allows the quantification of a number of DNA molecules at once. Droplet-digital PCR (ddPCR) can be applied to distinguish and quantify alterations with allele frequencies as low as 0.01% or less in cfDNA. Although short amplicons, less than 100 bp, can be amplified from formalin-fixed and paraffin-embedded (FFPE) samples by PCR analyses and fragmentation of DNA and crosslinking do not markedly affect the final results, it is obvious that fresh tumor sample are better 41-43. In addition, PCR is restricted by the requirement of prior knowledge of specific gene mutations or copy number variations to be analysed 22.

NGS can be used to detect tumor-specific gene mutations at frequencies as low as 0.05% in the whole genome or exome 44, 45. NGS can independently capture the complete panel of mutated genes in a cancer and is not based on prior knowledge of target genes. A hybrid approach incorporating droplet-digital PCR and NGS in liquid biopsy has been proposed to upgrade the sensitivity and specificity of the detection method 46.

Quantitative reverse-transcription PCR (RT-qPCR) is a fast, sensitive and low-throughput method, while microarray and deep sequencing are high-throughput assays that can be used to detect the whole transcriptome and non-coding RNAs in various types of samples including body fluids 47-49. Therefore, circulating tumor RNAs, including mRNAs, miRNAs, and lncRNAs, can be detected by RT-qPCR, microarray analysis and RNA-deep sequencing technology 49.

### Clinical applications of different ctNAs in sarcomas

The analysis of ctNAs allows for the detection of genetic and epigenetic variations in tumors over time. For sarcomas, genetic variations including mutational changes, translocations and fusions, have been identified from ctDNA in liquid biopsy 50, 51. Correspondingly, epigenetics variations in sarcomas mainly encompass three types: DNA methylation, histone modification and non-coding RNA modifications 52, 53. To date, only circulating non-coding RNAs , mainly microRNAs, have been studied in sarcoma liquid biopsy samples 38, 40, 54, 55. Previous studies have demonstrated that the levels of ctNAs in sarcomas are associated with tumorigenesis, progression and resistance to therapy 40, 46, 50, 54, 56-60. Therefore, ctNAs show the potential to serve as non-invasive biomarkers in monitoring sarcoma progression and therapeutic response.

A ctDNA detection assay was recently approved by the US Food and Drug Administration for use in lung cancer to identify the molecular composition of tumors 22. Tumor-derived mutated genes and fusion gene existed in many sarcoma subtypes 61, 62, and researches focusing on the potential of these mutated gene as non-invasive marker is ongoing 18, 50, 57, 59, 63-65.

miRNAs are a set of endogenous regulatory single-strand non-coding RNAs found in eukaryotes that are approximately 19-25 nucleotides in length 66. Recent studies have shown that miRNAs are involved in cell proliferation, differentiation and apoptosis as well as development and disease 67-73. Accumulating research has demonstrated that miRNAs are expressed in cell lines and tissue samples from bone and soft tissue sarcoma 58, 66, 73, 74, and merging evidence has indicated that circulating miRNAs can be detected in body fluids of cancer patients 40, 75, 76. Because of their stability in plasma or serum, miRNAs are considered promising biomarkers for early cancer diagnosis and prognosis prediction 40, 75, 76.

Below, we will discuss the current literature regarding the association of ctDNAs and ctRNAs, including mRNAs, miRNAs, and lncRNAs, and their clinical utility in various subtypes of sarcomas. All of the ctNAs identified thus far and their roles in sarcoma are summarized in Table 3.

### Osteosarcoma

Osteosarcoma (OS) is the most common malignant bone tumor and mainly affects the long bones of children and adolescents. In genetics, OS is associated with complex genomic alterations, including point mutations, deletions, amplifications and structural variants of various genes 77. The application of ctDNA in OS patients is limited. At the time of writing, only two studies evaluating liquid biopsy in OS have been performed 59, 64. In one study, the authors examined whether common somatic mutations could be detected in ctDNA from OS patient plasma 64. Mutations in *TP53, ATRX, DLG2* and *MET* in ctDNA were identified by targeted NGS in primary tissue samples and plasma samples, and these mutations were associated with the clinical course 64. In another study, copy number gains of chromosome arm 8q were detected in peripheral blood in OS patients 57. The changing level of ctDNA was correlated with tumor burden 59. An understanding of the clinical utility of ctDNA detection in this highly heterogeneous tumor is in the early stages, and further research is required.

Multiple circulating miRNAs have been detected that play oncogenic or antitumor-suppressor roles in OS. miR-542-3p was initially shown to enhance U2OS cell proliferation and migration 78. In a subsequent study, higher serum levels of miR-542-3p were detected in OS patients than in healthy individuals with sufficient sensitivity and specificity, and elevated miR-542-3p was associated with poor progression-free survival and overall survival 79. Other miRNAs, such as miR-221, miR-191, miR-421, and miR-124, have also been found in the circulation of OS patients and play oncogenic roles 80-83. Furthermore, miR-221, miR-191 and miR-421 levels were significantly upregulated in osteosarcoma tissues and serum compared with the respective levels in normal controls, however, miR-124 was remarkably decreased 80-83. These miRNAs may serve as diagnostic indicators or prognostic biomarkers in the future.

Owing to the application of neoadjuvant chemotherapy, the 5-year survival rate of OS patients after limb salvage surgery has reached 60%-70%, but the prognosis of patients with chemotherapy resistance is poor because of the lack of predictive biomarkers. Recently, Fujiwara et al. found that circulating miR-25-3p levels were inversely correlated with chemotherapy response in OS patients 74, 84. Simultaneously, they found that miR-25-3p induces chemoresistance via *Dickkopf WNT Signaling pathway inhibitor 3 (DKK3)* 74, 84. Moreover, elevated serum levels of miR-21 were also linked with chemo-resistance in OS patients and indicated a poor progression-free survival and overall survival 85. These miRNAs may be potential therapeutic targets for OS. Besides above, Allen-Rhoades et al reported that miR-214 is a prognostic marker in metastatic OS, and its level is negatively correlated with prognosis 86. This indicates that some miRNAs are worth studying as prognostic markers in OS patients.

In addition to oncogenic miRNAs, some studies have shown that other miRNAs act as tumor suppressors in OS. miR-491 binds to the 3′ untranslated region of *CRYAB* to mediate apoptosis in OS cells 87.The decreased level of miR-491 in OS patient serum was associated with lung metastasis, chemoresistance and a poor overall survival rate 87. miR-491 was an independent prognostic factor in OS patients and a potential biomarker in liquid biopsy. The levels of other miRNAs, such as miR-124, miR-101, miR-497 and miR-195, could be detected in peripheral blood samples and were downregulated in OS patients compared with the respective levels in healthy individuals 83, 88-91. Furthermore, these miRNAs are also related to higher pathological grade, metastasis, local recurrence and chemo-resistance 83, 88-91.

Some studies have shown that different combinations of miRNAs are associated with prognosis in OS. Decreased miR-133b or miR-206 alone in OS patient serum was associated with high tumor grade, metastasis, and recurrence 92. Moreover, a reduced level of miR-133b was linked with chemo-resistance and poor disease-free survival and overall survival in OS 92. However, a decrease in both miR-133b and miR-206 was correlated with a worse outcome than a decrease in miR-133b or miR-206 alone 92. Combinations of two or more circulating miRNAs have been used to predict the outcome in OS patients in other studies 93-96. Asano et al. recently analysed miRNA levels in the serum of 1,002 patients with bone and soft tissue tumors and formed an index that when combined with seven serum microRNAs can effectively distinguish patients with sarcoma from those with benign tumors, with a high sensitivity of 90% and a specificity of 95% 55. These results indicate that the use of combinations of miRNAs is a promising strategy for early and precise diagnosis and prediction of the prognosis of sarcoma in liquid biopsy samples.

LncRNAs are a group of non-protein-coding molecules of more than 200 nucleotides in length 97. LncRNAs are also involved in the development, proliferation, differentiation and apoptosis of cancer cells 98-100. Multiple studies have shown that lncRNAs play an important role in the regulation of progression and metastasis in many types of cancer, such as hepatocellular carcinoma 101, colorectal cancer 102-104 and breast cancer 105, 106.

The role of lncRNAs in sarcoma,especially in OS, has been the subject of investigation. Many lncRNAs participate in proliferation, differentiation, invasiveness, and even chemo-resistance in OS, and the levels of lncRNAs have been associated with clinicopathological features in OS patients 107-110.Therefore, circulating lncRNAs are attractive as potential noninvasive biomarkers. However, at the time of writing, only two studies have been reported. In 2016,Ma et al. showed that the plasma lncRNATUG1 was overexpressed in OS patients and that its level correlated with poor prognosis and disease status 111. The level of the circulating lncRNA UCA1 showed significant differences between OS patients and healthy individuals 112. Furthermore, compared with that in healthy individuals, the level of UCA1 expression in OS patients was higher, and the overall survival and disease-free survival were poorer 112. These studies suggest that the lncRNAs TUG1 and UCA1 could be potential non-invasive biomarkers for the diagnosis and prognosis of OS patients.

### Ewing sarcoma

Ewing sarcoma (ES) is a highly aggressive cancer that is predominantly founded in children and adolescents 113. ES is characterized by chromosomal translocation of the* EWS* gene to locations carrying different *ETS-*related genes 113, 114.Previous studies have indicated that ES has few recurrent somatic event besides *EWSR1-ETS* fusions 113, 115, 116. Only a few studies have indicated that mutations in *STAG2* and *Tp53* can be detected by NGS and are associated with a worse prognosis in ES 115-117. Therefore, reports on somatic events in liquid biopsy samples in ES are insufficient. Recently, two reports indicated that *STAG2* and *Tp53* mutations were found in ES patient peripheral blood samples 59, 118. Furthermore, they found that changes in the ctDNA level over time corresponded to response to therapy 59, 118.

Genetically, the most common gene fusions in ES cases are the *EWS-FLI1* (90%-95%) and *EWS-ERG* (5%-10%) gene fusions 113. The well-characterized genetic characteristics in ES provide the possibility to use liquid biopsy to monitor these gene fusion events. Hayashi et al. 63 found that circulating gene fusion levels paralleled with tumor burden and tumor metastasis in an ES xenotransplant mouse model, which prompted the authors to study the correlation between circulating gene fusion levels and tumor dynamics in ES patients. Subsequently, they found that the level of the *EWS-FLI1* fusion gene in the circulation decreased after chemotherapy or surgery and then started to rise when the tumor relapsed before clinical or radiographic evidence was confirmed 63. This result suggests that fusion genes can be used as non-invasive biomarkers for the prognosis and evaluation of therapeutic effects in ES patients. Although only three ES patients were analysed in the study, these findings suggest promising applications for liquid biopsy in ES. Accumulating evidence has shown that the level of the *EWSR1* fusion gene in the circulation decreases rapidly after initial chemotherapy in most patients and increases with tumor recurrence 18, 118. These results indicate that the levels of circulating *EWSR1-FLI1*and *EWSR1-ERG* in ES patients can reflect the tumor burden and relapse status 18, 63, 118. These studies further support the therapeutic application of the liquid biopsy strategies. Shulman and colleagues showed that ctDNA levels were inversely associated with disease-free survival and overall survival in a larger cohort of cancer patients, including 94 ES cases, indicating the potential of ctDNA as a non-invasive prognostic biomarker in clinical practice 59.

As early as 1997, a study showed that *EWS-FLI1* transcripts could be detected in peripheral blood and bone marrow samples from ES patients by RT-PCR 119. Although the exact physiological and clinical significance of circulating *EWS-FLI1* transcripts was not fully understood at the time, the results provided an important foundation for subsequent study. Alava et al. later demonstrated that changes in circulating *EWS-FLI1/ERG* transcript levels correlated with the disease status of ES patients 120. The circulating *EWS-FLI1/ERG* transcripts became undetectable after therapy and were then detectable before the disease progressed 120. Avigadet al. reported nearly identical results and showed that circulating mutated fusion gene transcripts in ES patients could predict prognosis 121. These studies can be regarded as early explorations into liquid biopsy in ES patients, and the results indicate that circulating tumor-specific fusion gene transcripts in peripheral blood can reflect the therapeutic effect in tumors and predict tumor progression. A recent study by Allegretti demonstrated that the number of plasma *EWS-FLI1* fusion gene transcripts paralleled the positron emission tomography volumetric parameters in ES patients, which provided preliminary evidence for using circulating gene transcripts to monitor the therapeutic efficacy of treatments in ES and evaluate the presence of residual lesions 122.

While the majority of reports have focused on mutated nuclear DNA transcripts as circulating biomarkers in ES, studies have shown that mutated mitochondrial DNA (mtDNA), an extra-chromosomal genome that replicates independently of nuclear DNA 123, 124, can be detected in many types of body fluids in patients with various cancers 123-125. Yu and collaborators demonstrated that the mtDNA level in the serum of ES patients was approximately two-fold lower than that of healthy adults, indicating the potential for mtDNA as a diagnostic marker to identify ES patients 126. In addition, the concentration of mtDNA in the serum was related to tumor metastasis in ES patients.

Compared with that in OS, research on circulating miRNAs in ES is rare. At the time of writing, only one study has been published, in which the authors examined circulating miR-125b in a group of Chinese ES patients 127. The results demonstrated that the circulating miR-125b level was decreased in the serum from ES patients compared with healthy individuals and was linked to poor response to chemotherapy. Previous research showed that miR-125b plays a tumor suppressor role in ES cells by targeting the *PI3K-AKT-mTOR* signalling pathway 128, and the chemoresistance function induced by miR-125b occurs via suppression of the apoptotic mediators *p53* and *BAK* 129. These results suggest that circulating miR-125b may be a non-invasive biomarker in ES patients and requires further validation in a larger cohort.

### Chondrosarcoma

Chondrosarcoma (CS) is the second most common primary bone malignancy. It represents a collection of heterogeneous bone cancers characterized by hyaline cartilaginous neoplastic tissue. Genetically, mutant isocitratedehydrogenase (IDH) 1 and 2 genes have been identified in more than half of central and periosteal CS cases 130, 131. Further studies found that the overall survival of CS patients with *IDH1/IDH2* mutations was significantly lower than that of patients without *IDH1/IDH2* mutations 132. Gutteridge et al. identified mutant *IDH* in the plasma of chondrosarcoma patients 133. The authors demonstrated that cell-free *IDH1/IDH2* could be detected in high-grade chondrosarcoma and dedifferentiated chondrosarcoma patients during the preoperative period; moreover, a high level of mutant *IDH* correlated with poor prognosis 133. Mutant *IDH1* was detected in plasma prior to traditional radiological imaging and clinical changes in patients whose tumors relapsed or metastasized during follow-up periods 133.These results indicate that mutated *IDH* in circulation may be a potential non-invasive biomarker to monitor chondrosarcoma patients in the clinical setting. Accumulating evidence has demonstrated that *the COL2A1* 134 and exostosin glycosyltransferase (*EXT1* or *EXT2*) genes are also frequently altered in CS 134, 135. Additionally, other sporadic mutations such as mutations in *TP53, pRB, AKT1, and MDM2* have also been identified in CS 134, 136, 137. However, the above altered genes have not been applied in liquid biopsy.

miRNAs are also involved in normal chondrogenesis 138, and some miRNAs play an oncogenic role in CS, such as miR-181a, which is overexpressed in high-grade CS and promotes the formation of vascular endothelial growth factor 139. In contrast, miR-100 is a tumor suppressor that is downregulated in CS 140. Other epigenetic alterations in CS including DNA methylation 138, such as hypermethylation of the promoter region of the gene encoding the transcription factor of *RUNX3* and prompted proliferation and inhibited apoptosis in CS tumor cells 141. However, epigenetics alterations in CS have not been used in liquid biopsy, and further research is needed.

Although chromosomal translocation is sporadic in CS, some fusion genes have been detected in CS, such as *EWSR1-NR4A3, RBP56-NR4A3*, and *TCF12-NR4A3,* which were detected in extraskeletal myxoid chondrosarcoma 138, 142-144. Additionally, the *HEY1-NCOA2* or*IRF2BP2-CDX1* fusion genes resulting from t(1;5)(q42;q32) also exists in mesenchymal chondrosarcoma 144, 145. To date, these fusion genes have not been applied in liquid biopsy.

### Liposarcoma

Liposarcoma ( LPS) is the most common soft tissue sarcoma (STS) in adults and account for approximately 25% of all STSs 146. LPS represents a heterogeneous group of adipocytic malignancies that are classified into four key subtypes: well-differentiated liposarcoma, dedifferentiated liposarcoma, myxoid liposarcoma, and pleomorphic liposarcoma. Subtypes can be distinguished by the presence of the *FUS-CHOP* fusion oncoprotein (myxoid LPS) and *MDM2* overexpression (well-differentiated liposarcoma and dedifferentiated liposarcoma) 146; therefore, the above mutations have the potential to be promising biomarkers in LPS. Recently, Braiget al. found that the levels of the breakpoint t(12:16) and *TERT C228T* mutation in plasma in myxoid liposarcomas were associated with tumor burden and tumor dynamics 60. Although mutant *TP53* is not a common somatic event 146, it has also been identified in plasma from dedifferentiated LPS patients during *HDM2* inhibitor therapy 147. *HDM2* interacts with wild-type *TP53* and subsequently inhibits*TP53* 148. The circulating mutant *TP53* level was increased in patients treated with the *HDM2* inhibitor compared with those treated with other therapies, and the levels correlated with tumor size 147. These results suggest that the circulating *TP53* mutation burden can be used to evaluate the tumor response to targeted therapy.

In addition, the level of miR-3613-3p was significantly upregulated in whole-blood samples from dedifferentiated liposarcoma patients, and it may potentially serve as an independent diagnostic biomarker to distinguish dedifferentiated liposarcoma patients from healthy individuals and lipoma patients 149.

### Synovial sarcoma

Synovial sarcoma(SS) is an aggressive soft tissue malignancy which characterized by the formation of the *SYT-SSX* fusion gene. Several researchers have attempted to validate the potential use of the circulating *SYT-SSX* fusion gene as a non-invasive biomarker in SS patients 65, 150-152. In 2001, Hashimoto et al. detected the *SYT-SSX* fusion gene in peripheral blood by nested PCR in an SS patient 150. Although only a single patient was analysed, these results suggest that the circulating *SYT-SSX* fusion gene may be a promising non-invasive biomarker for monitoring SS patients. Similar findings were shown in a gastric SS patient 151. Based on these findings, subsequent studies investigated the potential of the circulating *SYT-SSX* fusion gene in liquid biopsy of SS patients. However, existing evidence revealed that the presence of the circulating *SYT-SSX* fusion gene was an infrequent event in SS patients and that the circulating *SYT-SSX* fusion gene was not an ideal marker to monitor tumor dynamics 65, 152, 153. Therefore, the *SYT-SSX* fusion gene may not be a reliable circulating biomarker and further research is needed in SS patients. Other than translocation, SSs are mutationally quiet 154.

Recent studies have reported promising findings related to circulating miRNAs in SS. Uotani et al. evaluated miR-92b-3p in the serum of SS patients with a miRNA microarray assay and found that it was correlated with tumor burden and tumor dynamics 155. Fricke et al. identified a panel of upregulated miRNAs, including miR-99a-5p, miR-146b-5p, miR-148b-3p, miR-195-5p, miR-223-3p, miR-500b-3p and miR-505-3p, in peripheral whole-blood samples using a gene chip miRNA array coupled with qRT-PCR 56. The authors found that these miRNAs could be used as diagnostic biomarkers for distinguishing patients with SS from patients with other sarcoma subtypes and healthy controls (Table 4), and these miRNAs could also be used to monitor local recurrence and distant metastasis. This study not only distinguished a panel of miRNAs as biomarkers in liquid biopsy of SS patients but also provided a practical protocol to screen miRNAs in body fluid.

### Rhabdomyosarcoma

Rhabdomyosarcoma (RMS) is a small round cell malignant tumor that is frequently found in children. RMSs are mainly categorized into alveolar rhabdomyosarcoma (ARMS), embryonal rhabdomyosarcoma (ERMS) and pleomorphic rhabdomyosarcoma (PRMS) 156, 157. Up to 90% of ARMSs present with t(1;13)(p36;q15) or t(2;13)(q35;q14), which results in the fusion of the *PAX3* gene with *FOXO1* or the *PAX7* gene with *FOXO1,* respectively 156-158. The specific known translocations in ARMS cases provide potential markers to monitor tumor dynamics. To detect sarcoma-specific translocations in cfDNA, translocation-specific sarcoma sequencing assays have been developed, and the *PAX3/FOXO1* translocation was detected in all ARMS patients in research by Klega et al. 118. Previous studies have demonstrated that patients with ARMS with *PAX3/FOXO1* have worse outcomes than those without the translocation 157. Subsequently, the *PAX3-FOXO1* fusion was also identified in a plasma sample from a patient with ARMS and proved to be a reliable biomarker to detect early tumor progression and recurrence 159, 160.

Although the t(1;13)(p36;q15) and t(2;13) (q35;q14) translocations are rare in ERMS and PRMS, *PAX3*, *PAX7 and FOXO1* are frequently overexpressed 161. It is valuable to further explore the upregulated *PAX* and *FOXO1* in liquid biopsy samples.

In addition to fusion genes and overexpressed *PAX3*, *PAX7 and FOXO1,* serum miRNAs (miR-1, miR-133a, miR-133b and miR-206) have been identified in RMS cases 162. Additionally, miR-206 can be used as a diagnostic biomarker in distinguishing RMS from non-RMS tumors with a sensitivity of 1.0 and a specificity of 0.913 162.

### Leiomyosarcoma

Leiomyosarcoma (LMS) is a highly aggressive malignant neoplasm derived from smooth muscle tissue that makes up approximately 10% of STSs 163. LMS commonly affects the uterus or retroperitoneum but also occurs throughout the body 164. LMS presents with a highly complex karyotype with no specific mutations 52, 165. Therefore, liquid biopsy in LMS is rare, and a recent study by Hemming et al. found that a higher level of ctDNA, including a panel of genes with copy number variations, detected by ultra-low-passage whole-genome sequencing was correlated with tumor size and tumor progression in LMS 166. In addition to primary sarcoma, liquid biopsy has also been used in some metastatic sarcomas. Nicholas C. Eastley et al. identified 5 of 11 metastatic STS patients as having cancer-related mutations including mutations in *TP53*, *PIK3CA* and *HRAS* in plasma samples 50. In addition, they found that these mutations correlated with tumor burden 50. The 5 patients encompassed two patients with LMS and one patient each with undifferentiated pleomorphic sarcoma, soft tissue chondrosarcoma, and epithelioid angiosarcoma 50.

Increasing evidence indicates that the miRNA expression profile can be used to distinguish many tumor types, including sarcomas 54, 167. miR-1 and miR-113a/b which are regulators of myogenesis, were significantly overexpressed in LMS 58, 168. A study found that miR-21 was differentially expressed between LMS and leiomyoma 169. Additionally, Guled et al. indicated that a collection of serum miRNAs, including miR-199b-5p, miR-320a, miR-199a-3p, miR-126, and miR-22, were differentially expressed between LMS and undifferentiated pleomorphic sarcoma 170. These results indicated that these blood-borne miRNAs could be used in the differential diagnosis of different sarcoma subtypes. Additionally, further studies need to be performed to explore more miRNAs as diagnostic markers in sarcoma.

### Gastrointestinal Stromal Tumor

Gastrointestinal stromal tumor (GIST) is the most common gastrointestinal sarcoma with an incidence of 15-20 cases per million per year 54, 171. *KIT* or platelet-derived growth factor receptor alpha (*PDGFRA*) gain-of-function mutations 54, 171 are present in approximately 85-90% of GIST cases 172, 173. Specific mutations in GIST have the potential to be markers in liquid biopsy*.* The application of liquid biopsy in GIST was first reported at the 2013 ASCO Annual Meeting 174. In this study, Reichardt et al found 84% overall concordance between plasma and tumor tissue samples in the detection of primary *KIT* exon 9 and 11 mutations 174. In the same year, Maier et al evaluated 291 plasma samples from 38 GIST patients using 25 different allele-specific ligation PCR assays covering *KIT* and *PDGFRA* alterations 175. The authors found that ratio of mutant ctDNA to wild-type ctDNA* for KIT* and *PDGFRA* were positively correlated with disease status 175. For instance, the ctDNA ratio was significantly higher in samples from patients with active disease than in samples from patients incomplete remission without residual disease. Taken together, the above studies indicated that mutant *KIT* or *PDGFRA* can be detected in the plasma of GIST patients and that the concentration of ctDNA correlates with tumor burden and therapy response. These findings suggest that ctDNA (*KIT* or *PDGFRA*) has the potential to be a reliable non-invasive biomarker for monitoring GIST. Subsequently, Bauer and collaborators reported promising results on liquid biopsy of GIST at the 2015 ASCO Annual Meeting 176. They collected 30 plasma and 15 matched tumor samples from 22 metastatic GIST patients and identified 87 nonsynonymous *Kit* alterations in plasma samples by a custom-designed targeted sequencing panel for the Illumina MiSeq platform. Primary mutations and resistance mutations were found in 41% and 86% of GIST patients, respectively 176. Nearly at the same time, Kang et al analysed mutations in *KIT, PDGFRA* and *BRAF* in GIST patient plasma samples via NGS 177. They found that additional mutations in plasma emerged in GIST patients who were resistant to tyrosine kinase inhibitor (TKI) treatment 177. These results indicate that blood-derived ctDNA can be used as a non-invasive marker for the prediction of treatment response in GIST patients. In the wake of the above research, Wanda and collaborators isolated plasma ctDNA before and after imatinib treatment from 4 imatinib-resistant GIST patients 178. They found that a primary mutation in *c-KIT* exon11 mutated into secondary *KIT* exon 13 and 18 mutations 178. The results indicated that the detection of secondary *c-KIT* mutations in ctDNA could be used to predict anticancer effects in GIST. More recently, studies exploring the role of primary mutations in GISTs have been initiated. Kang and coworkers analysed paired plasma-tissue samples in primary GIST patients and revealed 72% concordance between them 179. In a subsequent study, Boonstra et al proved that ddPCR is an effective method to detect *KIT* exon 11 mutations in GISTs in both tumor tissue and ctDNA with a 100% specificity and a 77% sensitivity 180. Furthermore, they also demonstrated that the level of ctDNA negatively correlated with treatment response; for instance, a decrease in *KIT* exon ctDNA corresponded to lesion remission or stable disease on radiological examination 180. In summary, specific mutations, including mutations in *KIT* or *PDGFRA,* in GIST have been proven to be promising non-invasive biomarkers in liquid biopsy. To date, there are no reports on circulating non-coding RNAs in GIST, leaving many questions unanswered on their potential role in evaluating tumor dynamics and TKI therapy response 171.

### Other sarcoma subtypes

Although STSs are a heterogeneous group of malignant solid neoplasms that include approximately 50 subtypes**,** there are several subtypes of STSs that have been understudied in relation to liquid biopsy 167.

Fibrosarcoma comprises a rare subtype of STS derived from fibrous connective tissue 167, 181. Fibrosarcoma with a complex karotype lacks of specific alterations such as recurrent point mutations and chromosomal translocation; therefore, there are very few reports on ctDNA in liquid biopsy for fibrosarcoma. Research on miRNAs in fibrosarcoma is limited. The miR-29 family activates MMP-2 to play tumor-suppressive roles in the HT-1080 human fibrosarcoma cell line 182. Moreover, miRNA-520c and miRNA-373 have also been identified in the above cell line, and they activate the *Ras/ Raf/ MEK/ Erk* signalling pathway and *NF-kB* to promote the tumor cell migration and invasion 183. The application of these miRNAs in liquid biopsy in fibrosarcoma requires further validation.

Angiosarcoma is a highly aggressive malignancy that originates from vascular or lymphatic tissues 184. Due to the rarity of angiosarcoma, few comprehensive studies of genetic changes in angiosarcoma have been reported 184. The most common aberrations in angiosarcoma are mutations in *TP53* (29%-35%) and losses of *CDKN2A*(26%) 146, 185, 186. Accordingly, Murali et al reported that more than 50% of angiosarcomas carry some genetic alterations affecting the *MAPK* signalling pathway, including mutations in *HRAS, KRAS, NRAS, MAPK, BRAF* and *NF-1* or amplifications of*MAPK1/CRKL, CRAF* or* BRAF* 186. Moreover, mutations in *FLT4, PTPRB, PLCG1, CIC* and *KDR* were also detected in angiosarcoma 187. These karyotype aberrations in angiosarcoma have the potential to be monitored in ctDNA in the future. Additionally, many miRNAs were identified in angiosarcoma, and miR-515-3p and miR-517c may be the most valuable diagnostic biomarkers. Sarver and collaborators identified that the above miRNAs were overexpressed by 12-fold in angiosarcoma relative to GIST, undifferentiated pleomorphic sarcoma and epithelioid sarcoma 188. Furthermore, the miR-515-5p, miR-517a, miR-518b, miR-519a, miR-522 and miR-17-92 clusters are also upregulated in angiosarcoma 188, 189. Further functional validation of these miRNAs in liquid biopsy will be required.

Malignant peripheral nerve sheath tumors (MPNSTs) constitute a rare subtype of STS and arise in large peripheral nerves 61. Accumulating evidence has identified non-synonymous mutations of the polycomb regressive complex2 (*PRC2*) subunits *SUZ112* and* EED* in approximately 80% of MPNSTs 190, 191. Additionally, many miRNAs have been found to be overexpressed in MPNSTs, such as miR-210, miR-339-5p, miR-10b and the miR-199a/214 cluster 192-194. Further, more extensive studies in liquid biopsy need to be performed.

Undifferentiated pleomorphic sarcoma (UPS) is an aggressive subtype of STS that frequently occurs in adults over the age of 40 146, 181. Due to its complex genetic features and difficult diagnosis, genetic alterations in UPS are also missing 146. Recently, Demoret et al found that ctDNA comprehensive genomic profiling (CGP) of UPS was poorly concordant with solid tumor CGP 195. The data shows that approximately 30% (2/6) of subjects had complete concordance and 50% (3/6) of subjects had complete or partial concordance 195. Given the poor concordance between ctDNA CGP and tumor CGP in UPS, more extensive studies need to be performed. Guled et al investigated the differential expression level of microRNAs between LMS and UPS and found that miR-199-5p was overexpressed in UPS 170. In addition, miR-138 was overexpressed in UPS and was negatively correlated with distant metastasis-free survival 196. In summary, the application of ctNAs in liquid biopsy for sarcomas shows promise. However, further research in different subtypes of STSs will need to be performed in subsequent studies.

## Conclusions and Perspectives

Although the application of liquid biopsy in bone and soft tissue sarcoma is still in its infancy, the field has shown important progress worthy of discussion, especially regarding ctNAs. Moreover, accumulating evidence has indicated that circulating tumor non-coding RNAs are also promising biomarkers. A panel of sarcoma-specific mutated genes can be detected in blood circulation and used as a non-invasive biomarker to monitor sarcoma patients. Furthermore, many types of non-coding RNAs, especially circulating miRNAs, have been indicated as specific biomarkers for diagnosing sarcoma, predicting prognosis and revealing chemotherapy resistance. Together these studies demonstrate the utility of circulating tumor nucleic acids in bone and soft tissue sarcomas as promising non-invasive biomarkers. Sarcomas with specific genetic mutations or translocations, such as GIST, CS, ES and RMS, have potential to be monitored with ctDNA in liquid biopsy. Conversely, some sarcomas harbour complex and irregular genomic changes, such as OS, LMS, LPS and UPS, and circulating non-coding RNA seems to be a promising surrogate in liquid biopsy in these diseases. Additionally, other epigenetic alterations, such as DNA methylation and histone modification, also need to be explored in liquid biopsy in the future. Thus, liquid biopsy opens up a new approach for effective and real-time tumor monitoring.

Despite these achievements, the application of ctNAs is still in the preclinical stage. Many studies include only few samples, and randomized clinical trials to support the preliminary results are lacking. Moreover, the sample and detection methods are inconsistent among different studies. Furthermore, the sensitivity and specificity of ctDNA as a biomarkers in liquid biopsy of peripheral blood are lower than expected. Further studies are required to explore the value of ctNAs in sarcoma patients.

## Figures and Tables

**Table 1:** Comparison of tissue biopsy and liquid biopsy in sarcoma

	Advantages	Limitations
**Tissue biopsy**	1. The gold standard of diagnosis, classification and staging of sarcoma 12, 13 2. Guides treatment in sarcoma 12, 13 3. Well-developed technical process and widely used in the clinic 12, 13	1. Invasive procedure 14, 15 2. Difficult to detect intratumoral and intertumoral heterogeneity with a single sample from a single site 16, 17 3. Possible clinical complications associated with tissue biopsy 14, 15 4. Difficult to sample certain parts of the body 12, 13 5. Difficult to sample continuously during the follow-up period 12, 13
**Liquid biopsy**	1. Non-invasive procedure 19, 22 2. Easily repeated sampling during the follow-up period 19, 22 3. Body fluid samples can reflect a panoramic view of the tumor in real time, including the primary and metastatic sites 19, 22 4. Dynamic evaluation of therapeutic effects 19, 22 5. Formation of personalized therapy based on tumor heterogeneity 19, 22 6. Easy sampling and not limited by the anatomic location of the tumor 19, 22	1. Difficult to find sensitive and specific biomarkers due to the high heterogeneity of sarcoma 13, 16, 195 2. Significance of biomarkers and clinicopathological features in sarcomas have not been fully established 16, 195 3. Differences between samples and sampling processes lead to poor consistency in results between different studies 16, 195

**Table 2:** Comparison of three types of liquid biopsy molecules (CTCs, ctNAs and exosomes)

Type	Source	Enrichmental or analytical methods	Strength	Limitation
**CTCs**	Peripheral blood	Physical property-based methods: tumor cell size 29, 197; biological property-based methods: mesenchymal cell markers, specific chromosomal translocations, specific gene mutation 24, 197	Non-invasive 29, 197; DNAs, RNAs and proteins can be analyzed and can be used as diagnostic or prognostic biomarkers 29, 197; can be used to evaluate the response to therapy and for early detection of residual and recurrent lesions 29, 197	Impact of heterogeneity on various CTC enrichment methods 24; low concentration of CTCs 24; absence of specific markers expressed by sarcoma tumor cells and highly heterogeneous phenotype 24
**ctDNA and** **ctRNA**	Serum or plasma	PCR(e.g., conventional RT-qPCR; dPCR; ddPCR); NGS; RNA-seq 16, 58	Non-invasive 16, 58; Can be used to analyze the tumor molecular heterogeneity; for precision therapeutic decision making based on specific gene mutation; for monitoring tumor burden and prognosis 16, 58; for early detection of emerging chemotherapy resistance 16, 58	Sample type and preparation protocols are different in different studies 16, 195; the relation between ctNAs and clinicopathological features is unclear 16, 195; sequencing data require professional bioinformatics analysis 16
**Exosomes**	Serum, plasma or other body fluids	Ultracentrifugation; density gradient centrifugation; membrane filtration; size exclusion chromatography; microfludic technologies 16, 58	Non-invasive; abundant in multiple body fluids; exosomes contain DNAs, RNAs and proteins that have diagnostic and prognostic value 16, 58	The purity of tumor-derived exosomes is different for different isolation methods; identification of the exosomes is difficult due to the lack of specific markers 16, 58

Abbreviations: CTCs: circulating tumor cells; ctNAs: circulating tumor nucleic acids, including circulating tumor DNA(ctDNA), specific gene transcripts, non-coding RNA; NGS: next generation sequence; RT-PCR: real time polymerase chain reaction; dPCR: digital polymerase chain reaction; ddPCR: droplet digital polymerase chain reaction.

**Table 3:** Summary of circulating tumor nucleic acids in bone and soft tissue sarcomas.

Sarcoma subtype	Circulating nucleic acid	Molecular target	Analysis method	Sample	Diagnostic value	Prognostic assessment	Response to therapy	Reference
OS	ctDNA	*TP53,ATRX,DLG2,*and*MET* mutations	tNGS	Plasma		√		64
		Chrosome 8q	ddPCR+NGS	Plasma		√		59
OS	ctRNA	miR-542-3p	RT-qPCR	Serum	√	√		79
		miR-221	RT-qPCR	Serum	√	√		80
		miR-191	RT-qPCR	Serum	√	√		81
		miR-124	RT-qPCR	Serum	√	√		83
		miR-421	RT-qPCR	Serum	√	√	√	82
		miR-25-3p	RT-qPCR	Serum	√	√	√	84
		miR-21	RT-qPCR	Serum		√	√	85
		miR-491	RT-qPCR	Serum		√		87
		miR-101	RT-qPCR	Serum		√		89
		miR-497	RT-qPCR	Serum		√		90
OS	ctRNA	miR-195	RT-qPCR	Serum		√	√	88
		miR-375	RT-qPCR	Serum		√	√	91
		miR133b and miR-206	RT-qPCR	Serum	√	√	√	92
		miR199a-3p, miR-21 and miR-143	RT-qPCR	Serum	√	√		93
		miR-215-5p andmiR-642a-5p	RT-qPCR	Serum		√		94
		miR-21, miR-221 and miR-106a	RT-qPCR	Serum		√		95
		miR-106a-5p, miR16-5p, miR-20a-5p, miR-425-5p, miR451a, miR-25-3P and miR139-5p	RT-qPCR	Serum		√	√	96
OS	ctRNA	lncRNA TUG1	RT-qPCR	Whole blood		√		111
		LncRNA UCA1	RT-qPCR	Plasma		√		112
ES	ctDNA	STAG2 and TP53	NGS	Plasma			√	59, 118
		*EWS/FLI* and *EWS/ERG* fusion sequences	dPCR	Plasma		√	√	18, 59, 63
		mtDNA	RT-qPCR	Serum	√	√		126
ES	ctRNA	*EWS/FLI1* and *EWS/ERG* transcripts	RT-qPCR	Plasma		√	√	119-122
		miR-125b	RT-qPCR	Serum	√			127
CS	ctDNA	mutant *IDH1/IDH2*	dPCR	Plasma	√	√		133
LPS	ctDNA	*TERT C228T* break point t(12:16)	qPCR	Plasma		√		60
		*TP53*	NGS	Plasma			√	147
	ctRNA	miR-3613-3p	RT-qPCR	Whole blood		√		149
SS	ctDNA	*SYT-SSX* fusion sequence	Nested PCR	Peripheral blood			√	150-152
	ctRNA	miR-92b-3p	RT-qPCR	Serum	√	√	√	155
		miR-99a-5p, miR-146b-5p, miR-148b-3p, miR-195-5p, miR-223-3p, miR-500b-3p and miR-505-3p	RT-qPCR	Serum		√	√	56
RMS	ctDNA	*PAX3-FOXO1* fusion	ddPCR	Plasma		√	√	159, 160
		miR-1, miR-133a, miR-133b and miR-206	RT-qPCR	Serum	√			162
LMS	ctDNA	*TP53, RB1* and *PTEN*	ULP-WGS	Plasma		√		166
	ctDNA	*TP53, PIK3CA* and *HRAS*	tNGS	Plasma		√		50
	ctRNA	miR-199b-5p, miR-320a, miR-199a-3p, miR-126, andmiR-22	RT-qPCR	Plasma	√	√		170
GIST	ctDNA	*KIT* or *PDGFRA*	L-PCR or dPCR	Plasma	√	√	√	174-180

Abbreviations: tNGS: targeted next generation sequencing; cNGS: custom next generation sequencing; L-NGS: ligation-next generation sequencing; ULP-WGS: ultra-low passage whole-genome sequencing; ddPCR: droplet-digital polymerase chain reaction; dPCR: digital polymerase chain reaction; RT-qPCR: real time quantitative polymerase chain reaction; L-PCR: ligation-polymerase chain reaction

**Table 4:** Expression level of miRNAs in active synovial sarcoma, synovial sarcoma with complete remission, healthy controls and others sarcoma subtypes

miRNA associated with active SS (upregulated)	SS with CR	Healthy control	Active LMS	Active MPNST	Active ES	Active LPS
hsa-miR-99a-5p	√	√		√		√
hsa-miR-146b-5p	√	√	√	√	√	√
hsa-miR-148b-3p	√	√	√	√	√	√
hsa-miR-195-5p	√	√				√
hsa-miR-223-3p	√	√	√	√	√	√
hsa-miR-500b-3p	√	√	√	√	√	√
hsa-miR-505-3p	√	√	√	√	√	√

Abbreviation: CR: complete remission; LMS: leiomyosarcoma; MPNST: malignant peripheral nerve sheath tumor;ES: Ewing sarcoma; LPS: liposarcoma

## Abbreviations

OS: osteosarcoma
ES: ewing sarcoma
CS: chondrosarcoma
LPS: liposarcoma
SS: synovial sarcoma
RMS: rhabdomyosarcoma
ARMS: alveolar rhabdomyosarcoma
ERMS: embryonal rhabdomyosarcoma
PRMS: pleomorphic rhabdomyosarcoma
LMS: leiomyosarcoma
GIST: gastrointestinal stromal tumor
MPNST: malignant peripheral nerve sheath tumor
UPS: undifferentiated pleomorphic sarcoma
STS: soft tissue sarcoma
ctNAs: circulating tumor nucleic acids
cfDNA: cell-free DNA
ctDNA: circulating tumor DNA
mtDNA: mitochondrial DNA
CTCs: circulating tumor cells
lncRNA: long non-coding RNA
PCR: polymerase chain reaction
dPCR: digital polymerase chain reaction
ddPCR: droplet digital polymerase chain reaction
RT-qPCR: quantitative reverse-transcription polymerase chain reaction
NGS: next generation sequencing
IDH: isocitrate dehydrogenase
EXT: exostosin glycosyltransferase
PDGFRA: plate-derived growth factor receptor alpha
TKIs: tyrosine kinase inhibitors
PRC2: polycomb regressive complex 2
